# MRI-Based Bladder Cancer Staging via YOLOv11 Segmentation and Deep Learning Classification

**DOI:** 10.3390/diseases14020045

**Published:** 2026-01-28

**Authors:** Phisit Katongtung, Kanokwatt Shiangjen, Watcharaporn Cholamjiak, Krittin Naravejsakul

**Affiliations:** 1 School of Information and Communication Technology, University of Phayao, Phayao 56000, Thailand; 65024333@up.ac.th (P.K.); kanokwatt.sh@up.ac.th (K.S.); 2 School of Science, University of Phayao, Phayao 56000, Thailand; watcharaporn.ch@up.ac.th; 3 School of Medicine, University of Phayao, Phayao 56000, Thailand

**Keywords:** bladder cancer staging, MRI, hybrid segmentation, deep learning, radiology-oriented workflow support

## Abstract

Background: Accurate staging of bladder cancer is critical for guiding clinical management, particularly the distinction between non–muscle-invasive (T1) and muscle-invasive (T2–T4) disease. Although MRI offers superior soft-tissue contrast, image interpretation remains opera-tor-dependent and subject to inter-observer variability. This study proposes an automated deep learning framework for MRI-based bladder cancer staging to support standardized radio-logical interpretation. Methods: A sequential AI-based pipeline was developed, integrating hybrid tumor segmentation using YOLOv11 for lesion detection and DeepLabV3 for boundary refinement, followed by three deep learning classifiers (VGG19, ResNet50, and Vision Transformer) for MRI-based stage prediction. A total of 416 T2-weighted MRI images with radiology-derived stage labels (T1–T4) were included, with data augmentation applied during training. Model performance was evaluated using accuracy, precision, recall, F1-score, and multi-class AUC. Performance un-certainty was characterized using patient-level bootstrap confidence intervals under a fixed training and evaluation pipeline. Results: All evaluated models demonstrated high and broadly comparable discriminative performance for MRI-based bladder cancer staging within the present dataset, with high point estimates of accuracy and AUC, particularly for differentiating non–muscle-invasive from muscle-invasive disease. Calibration analysis characterized the probabilistic behavior of predicted stage probabilities under the current experimental setting. Conclusions: The proposed framework demonstrates the feasibility of automated MRI-based bladder cancer staging derived from radiological reference labels and supports the potential of deep learning for stand-ardizing and reproducing MRI-based staging procedures. Rather than serving as an independent clinical decision-support system, the framework is intended as a methodological and work-flow-oriented tool for automated staging consistency. Further validation using multi-center datasets, patient-level data splitting prior to augmentation, pathology-confirmed reference stand-ards, and explainable AI techniques is required to establish generalizability and clinical relevance.

## 1. Introduction

Bladder cancer remains a major global health burden, consistently ranking among the ten most frequently diagnosed malignancies worldwide [1,2]. GLOBOCAN 2022 estimates indicate approximately 600,000 new cases and 220,000 deaths annually [1]. In the United States, the age-adjusted incidence rate is approximately 18 per 100,000 persons per year, with men disproportionately affected [3]. Despite therapeutic advances, outcome improvements, particularly in muscle-invasive bladder cancer (MIBC), have been modest, underscoring the need for earlier and more accurate staging to guide personalized therapy [3].

Accurate staging is critical for treatment selection. Non-muscle-invasive bladder cancer (NMIBC: Tis/Ta/T1) is typically managed with transurethral resection of bladder tumor (TURBT) and intravesical therapy, while muscle-invasive disease (MIBC: T2+) requires radical cystectomy, neoadjuvant chemotherapy, or multimodal therapy [4,5,6,7,8]. The distinction between T1 and T2 stages represents a pivotal decision point, as treatment intensity and prognosis differ substantially [4,6]. However, cystoscopy and TURBT-based staging workflows are limited by sampling error, inter-observer variability, and patient burden [9,10].

Multiparametric MRI (mp-MRI) offers superior soft-tissue contrast for local staging and assessment of detrusor and perivesical invasion [8,11,12,13]. The Vesical Imaging-Reporting and Data System (VI-RADS) was developed to standardize mp-MRI acquisition and interpretation, aiming to reduce variability and improve diagnostic communication [4,5,7]. Despite these advances, MRI interpretation remains time-consuming and reader-dependent. Classical dynamic MRI studies reported staging accuracy of approximately 62% with 32% overstaging, highlighting persistent challenges in reproducible interpretation [14].

Over the past decade, artificial intelligence (AI), particularly deep learning (DL), has transformed oncologic imaging through automated feature extraction, segmentation, and classification [15,16,17]. A recent systematic review and meta-analysis of image-based AI for MIBC prediction reported strong pooled performance for MRI-based DL models (AUC ≈ 0.9), while noting methodological heterogeneity and limited clinical translation [18]. DL-based segmentation studies have demonstrated feasible multi-region bladder wall and tumor delineation on MRI with promising Dice scores, establishing segmentation as a critical prerequisite for accurate staging [19,20]. However, most studies remain retrospective and single-center, with limited prospective or multi-institutional validation and variable standardization, key barriers to clinical adoption [17,18].

Multi-scale feature extraction has been widely adopted in medical image segmentation to capture contextual information across different spatial resolutions, as demonstrated in recent architectures such as MB-TaylorFormer v2 [21], ESTINet [22], and DDMSNet [23]. Rather than introducing a novel segmentation architecture, this study builds upon these established principles to explore their integration within a clinically oriented, end-to-end analytical pipeline. We hypothesize that coupling robust tumor localization and segmentation with downstream stage classification can reduce background interference, focus feature learning on anatomically relevant regions, and produce outputs aligned with clinically meaningful decision points based on tumor invasion depth (T1–T4).

Guided by the Vesical Imaging Reporting and Data System (VI-RADS) framework [4,5,6,7] and contemporary multiparametric MRI (mp-MRI) practice [8,11,12,13], we developed an automated MRI-based bladder cancer staging framework that integrates hybrid tumor segmentation—using YOLOv11 for detection and DeepLabV3 for boundary refinement—with multi-model stage classification employing VGG19, ResNet50, and a Vision Transformer. This modular and sequential design emphasizes reproducibility, interpretability, and computational efficiency, with the goal of supporting integration into routine radiological workflows rather than replacing expert clinical judgment.

The objectives of this study are threefold: (1) to develop and validate a hybrid segmentation model (YOLOv11 and DeepLabV3) for automatic bladder tumor identification and delineation on MRI; (2) to train and compare three deep learning classifiers (VGG19, ResNet50, and Vision Transformer) for predicting MRI-based tumor stage categories (T1–T4) derived from VI-RADS–guided imaging assessment using segmented regions of interest; and (3) to evaluate the translational potential of this automated pipeline as a radiology-oriented clinical decision-support tool for standardized MRI-based staging, contextualized within VI-RADS guidelines and previously reported AI performance in bladder cancer imaging [4,5,6,7,8,11,18].

## 2. Materials and Methods

### 2.1. MRI Data Acquisition and Preprocessing

The MRI dataset used in this study was obtained from the Bladder Cancer Classification dataset publicly available on the Kaggle platform [24,25]. The dataset initially consisted of 1285, T2-weighted bladder MRI images, categorized into two clinical labels: muscle-invasive bladder cancer (MIBC) and non–muscle-invasive bladder cancer (NMIBC). Each image was independently reviewed, verified, and certified by two board-certified radiologists and one oncologist to ensure diagnostic validity, proper labeling, and clinical relevance. After this expert verification process, 416 high-quality MRI scans were retained for deep-learning model development. Each patient contributed multiple MRI slices to the dataset. To avoid within-subject data leakage and account for intra-patient correlation, all data splitting was performed at the patient level, ensuring that slices from the same patient were assigned exclusively to either the training or test set.

Although the original Kaggle dataset provides only binary labels (MIBC vs. NMIBC), a finer-grained MRI-based stage categorization was derived in this study using the Vesical Imaging Reporting and Data System (VI-RADS) criteria [4,6]. VI-RADS is a standardized radiological scoring system designed to assess the likelihood and extent of muscle invasion based on multiparametric MRI findings. Accordingly, imaging features such as detrusor muscle involvement, perivesical fat infiltration, and extravesical extension were evaluated to assign each case to one of four MRI-derived tumor stage categories (T1–T4). These stage labels represent imaging-based stage assignments inferred from MRI interpretation rather than pathology-confirmed T staging. The MRI-based stage categories were defined as follows:

T1: Tumor confined to the subepithelial connective tissue

T2: Tumor invading the detrusor muscle

T3: Tumor extending into the perivesical tissue

T4: Tumor invading adjacent organs (e.g., prostate, uterus, or pelvic wall)

This refined staging framework enables more clinically meaningful analysis and provides better insight into tumor progression and treatment planning, since accurate identification of muscle invasion is critical for determining therapeutic strategies such as transurethral resection vs. radical cystectomy.

After expert staging, the data distribution comprised T1 (272 images), T2 (76 images), T3 (41 images), and T4 (27 images), revealing a severe class imbalance among advanced stages. To overcome this limitation, an extensive data-augmentation strategy was applied, including random rotations (±20°), horizontal/vertical flips, brightness and contrast adjustments, Gaussian noise injection, and elastic deformations. These operations enhanced image diversity while preserving bladder anatomy and tumor integrity. The augmented dataset expanded to 1610 images, providing more balanced class representation, as summarized in Table 1.

All MRI images were resized to 512 × 512 pixels and normalized to the intensity range [0, 1]. Data augmentation was applied to the original MRI slices prior to dataset splitting in order to increase sample diversity, using standard geometric transformations. The augmented dataset was subsequently divided into training (80%) and testing (20%) subsets.

This preprocessing pipeline ensured consistent image dimensions and intensity scaling to facilitate stable model training. In this study, data augmentation was applied prior to dataset splitting, and augmented images were included in both the training and test sets. As a result, the evaluation reflects model performance under augmented imaging conditions rather than performance exclusively on original MRI images.

We acknowledge that performing data augmentation before the train–test split may introduce correlations between augmented samples derived from the same original image, potentially leading to optimistic performance estimates. This methodological limitation has been explicitly considered in the interpretation of the reported results.

### 2.2. YOLOv11-Based Tumor Segmentation Framework

The segmentation process aimed to accurately localize and delineate bladder tumor regions from T2-weighted MRI images. Manual labeling of tumor masses was first performed using the Roboflow platform [26], where each image was annotated by medical imaging specialists to define the tumor boundaries. The annotated dataset was subsequently used to train a YOLOv11 segmentation model, fine-tuned with hyperparameters optimized for medical image analysis (learning rate = 0.001, batch size = 16, epochs = 30).

The YOLOv11 model efficiently identifies and segments tumor masses by leveraging its multi-scale feature extraction and bounding polygon output layers. This model combines a backbone encoder–decoder architecture with dilated convolutions and multi-branch context modules, allowing it to capture both local texture and global context features critical for accurate delineation of bladder wall invasion. To further refine segmentation accuracy, the YOLOv11 output was integrated with a DeepLabV3 post-processing stage [27], which applies atrous spatial pyramid pooling (ASPP) to enhance boundary precision and reduce background noise. This hybrid approach effectively suppressed irrelevant anatomical structures while emphasizing tumor-containing regions. In this study, the segmentation component was primarily used as a functional localization step to generate regions of interest for downstream classification, rather than being evaluated as an independent segmentation benchmark.

For tumor localization, YOLOv11 outputs multiple candidate bounding boxes per MRI slice, each associated with a confidence score. A confidence threshold of 0.5 was applied to filter low-confidence detections. In cases where multiple bounding boxes exceeded the threshold, the bounding box with the highest confidence score was selected as the tumor region of interest (ROI).

If no detection exceeded the predefined threshold, the bounding box with the highest confidence score was retained to avoid missing tumor regions. This confidence-based selection strategy ensured consistent ROI extraction for subsequent segmentation and classification.

**Figure 1:**
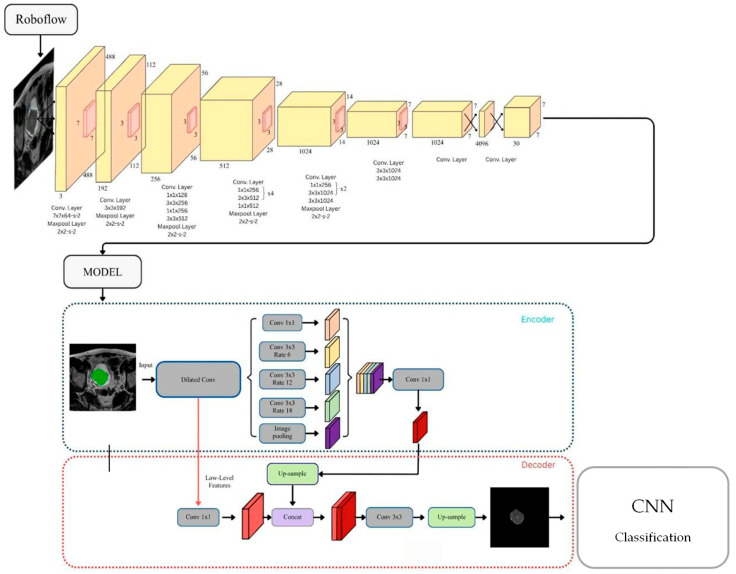
Overview of the YOLOv11 and DeepLabV3 bladder tumor segmentation framework.

After segmentation, the model generated binary masks for each image, which were used to automatically extract cropped tumor regions (Cropped Tumors). These cropped ROIs served as standardized inputs for the subsequent classification stage (T1–T4 staging model). Figure 1 provides an overview of the bladder tumor segmentation framework, illustrating the annotation process in Roboflow, the YOLOv11 and DeepLabV3 architectures, and the final ROI extraction results. To address the issue of limited sample size and class imbalance, data augmentation techniques [28] were applied to the segmented tumor images. The augmentation pipeline included random rotation (±15°), translation (horizontal/vertical ±10%), shear deformation (±0.1), and zoom in/out (±10%). As a result, the dataset expanded to 1610 tumor images, comprising T1: 531, T2: 372, T3: 365, and T4: 342 images (see Table 1). This augmentation enhanced the model’s robustness and mitigated overfitting by providing greater intra-class variability.

The framework begins with manual annotation of MRI bladder tumor masses using the Roboflow platform. The labeled dataset is then used to train the YOLOv11 segmentation network, which integrates DeepLabV3 contextual refinement to improve boundary accuracy and noise suppression. The resulting binary masks are used to extract cropped tumor regions (Cropped Tumors) for subsequent classification into T1–T4 stages.

### 2.3. Deep Learning Classification for Stage Prediction

The classification stage represents the second component of the proposed AI-based framework for MRI-based bladder cancer staging. After segmentation by the YOLOv11 and DeepLabV3 models, each MRI image was cropped to isolate the Region of Interest (ROI) containing the bladder tumor. These ROI images were normalized and used as input for several deep learning–based classifiers designed to predict the pathological stage of the tumor. The following three convolutional and transformer architectures were implemented for comparative analysis: VGG19 [29], ResNet50 [30], and Vision Transformer (ViT) [31]. Each model was trained to classify tumors into four pathological stages (T1, T2, T3, and T4), representing progressively deeper muscular and extravesical invasion. All models were trained under identical experimental settings using categorical cross-entropy loss and optimized with the Adam optimizer (learning rate = 0.0001, batch size = 32). The dataset was augmented (see Section 2.1) to address class imbalance and divided into training (80%) and testing (20%) sets. Early stopping and dropout regularization were applied to prevent overfitting and ensure convergence stability.

The Vision Transformer (ViT) model was included to explore the potential of attention feature extraction in comparison with conventional Convolutional Neural Network (CNN) architectures. By partitioning MRI images into non-overlapping patches and processing them through a transformer encoder, ViT effectively captures global contextual dependencies that may reflect subtle textural and morphological differences across tumor stages. This comparative framework enables a robust evaluation of both CNN and transformer models for bladder cancer staging. The integration of segmentation-guided ROI extraction and deep learning classification provides an interpretable and scalable AI pipeline that supports radiologists in accurate stage assessment and treatment planning.

### 2.4. Research Workflow

Model performance was quantitatively evaluated using accuracy, precision, recall, F1-score, and multi-class AUC metrics. The final interpretation focuses on the feasibility of the proposed framework for radiology-oriented analysis and standardized replication of MRI-based staging derived from established imaging criteria, rather than as an independent clinical decision-support system.

**Figure 2:**
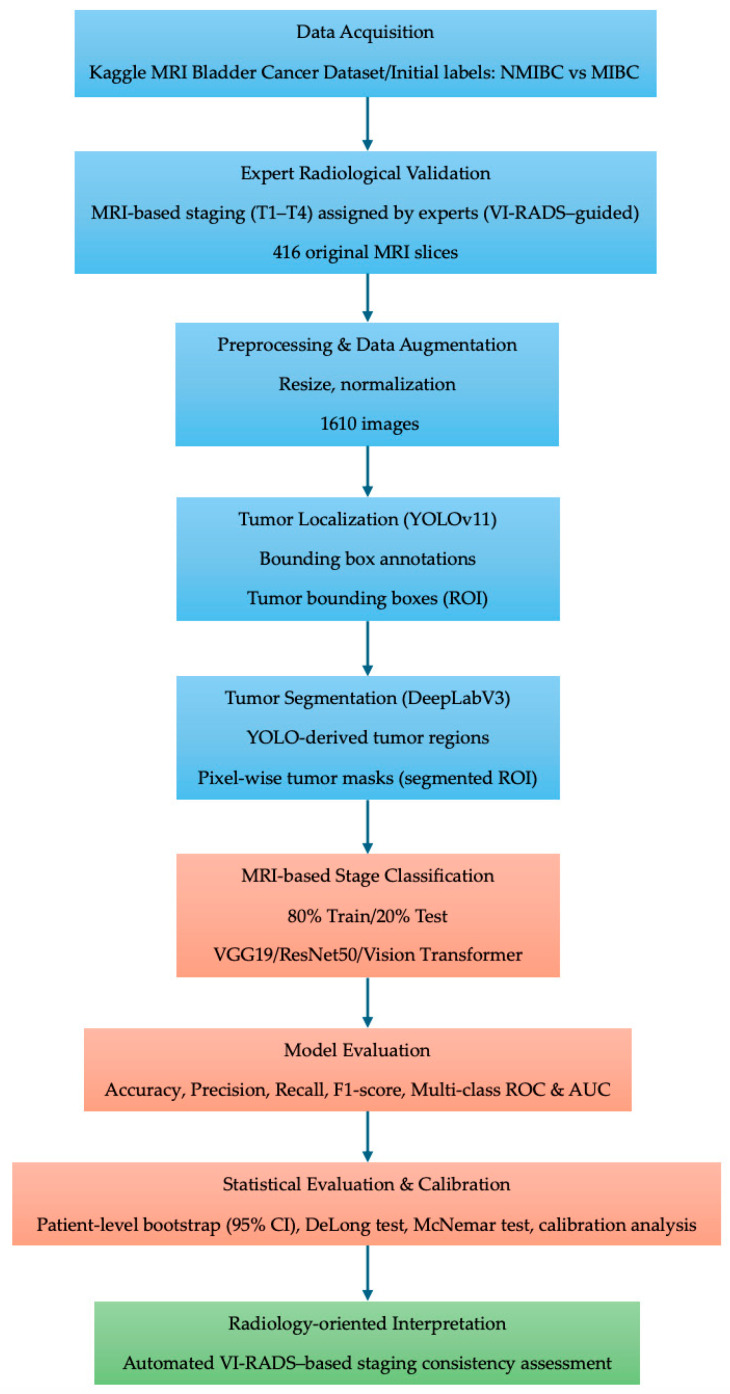
Overview of the proposed sequential deep learning workflow for MRI-based bladder cancer staging. The pipeline consists of expert-validated MRI-based stage assignment, preprocessing and data augmentation, tumor localization using YOLOv11, YOLO-guided tumor segmentation using DeepLabV3, and subsequent stage classification using CNN- and Transformer-based models. Performance evaluation was conducted using standard classification metrics and multi-class ROC analysis.

Bootstrap resampling was applied at the evaluation stage to characterize the uncertainty of performance estimates under the fixed trained pipeline. All preprocessing, ROI extraction, and segmentation steps were held constant during resampling, and the bootstrap analysis was not intended to represent end-to-end re-training of the entire data-generating and learning process. Accordingly, the resulting confidence intervals describe sampling variability conditional on the adopted experimental design rather than generalization beyond the evaluated dataset. An overview of the complete research workflow is illustrated in Figure 2.

## 3. Results

This section presents a comparative evaluation of the deep learning models used for MRI-based bladder cancer stage prediction. All models were evaluated on the same dataset, consisting of cropped Regions of Interest (ROIs) obtained from the segmentation stage. Performance metrics, including accuracy, precision, recall, and F1-score, were computed for each MRI-derived tumor stage (T1–T4) to characterize classification behavior across disease severities under a common experimental setting.

Across the evaluated architectures, VGG19, ResNet50, and Vision Transformer (ViT) demonstrated broadly comparable performance in differentiating bladder cancer stages within the present dataset. While ResNet50 yielded higher point estimates of overall accuracy under the adopted training and evaluation pipeline, these differences were modest and should be interpreted as descriptive rather than indicative of general or population-level superiority. VGG19 showed consistent classification behavior across stages, and ViT achieved competitive performance despite the relatively limited sample size typical of MRI-based datasets.

The detailed comparative results, including stage-wise performance metrics and model-specific observations, are summarized in Table 2 and are intended to provide a conditional comparison of model behavior under the fixed dataset and pipeline considered in this study.

The comparative analysis across three deep learning architectures—VGG19, ResNet50, and Vision Transformer (ViT)—shows that all models achieved high and comparable performance for multi-class MRI-based bladder cancer stage prediction within the present dataset. While ResNet50 yielded higher point estimates for several metrics, these differences should be interpreted as descriptive and conditional on the adopted experimental setting, rather than as evidence of general or clinical superiority.

**Figure 3:**
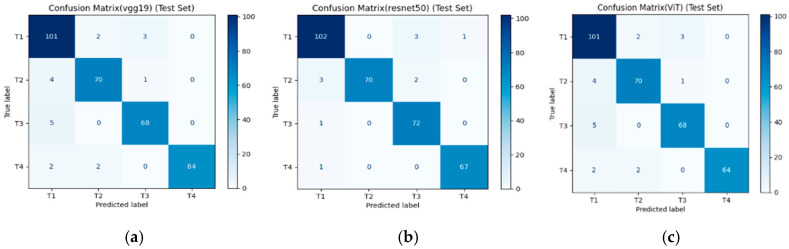
Confusion matrices illustrating the classification performance of three deep learning models—VGG19 (**a**), ResNet50 (**b**), and ViT (**c**)—for bladder cancer stage prediction using MRI test data.

From Figure 3, each matrix presents the distribution of true versus predicted labels across the four MRI-based tumor stage categories (T1–T4). Within the present dataset and experimental setting, the ResNet50 model shows comparatively fewer misclassifications in differentiating between intermediate and advanced stages (T2–T3 and T3–T4). In contrast, VGG19 and Vision Transformer (ViT) exhibit similar overall classification patterns but display slightly higher confusion between early-stage categories (T1–T2), which may reflect challenges in capturing subtle imaging features associated with early muscular invasion. These observations are descriptive and conditional on the adopted dataset and evaluation pipeline, and should not be interpreted as evidence of general or population-level superiority among architectures.

**Figure 4:**
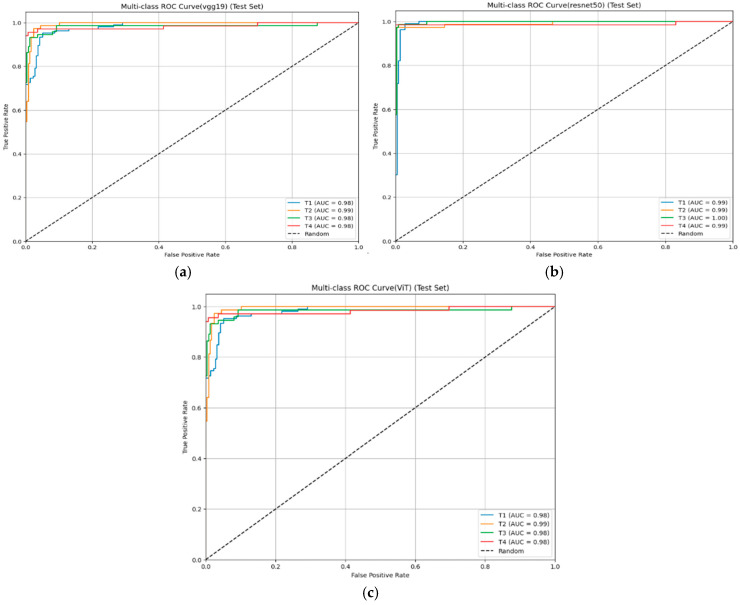
Multi-class Receiver Operating Characteristic (ROC) curves comparing the stage-wise classification performance of the three deep learning models: VGG19 (**a**), ResNet50 (**b**), and ViT (**c**) on the MRI test set.

From Figure 4, each curve represents the model’s ability to distinguish among four pathological stages of bladder cancer (T1–T4), with the Area Under the Curve (AUC) values reported for each stage. All models achieved high discriminative capability (AUC > 0.90 across all stages), indicating excellent sensitivity–specificity balance. Among them, ResNet50 achieved the highest and most consistent AUC values (T1–T4 ≈ 0.99), demonstrating superior robustness and generalization across both early and advanced tumor stages. In contrast, VGG19 and ViT also showed strong classification performance (AUC ≈ 0.98), confirming the reliability of both CNN and transformer architectures for MRI-based bladder cancer staging.

**Figure 5:**
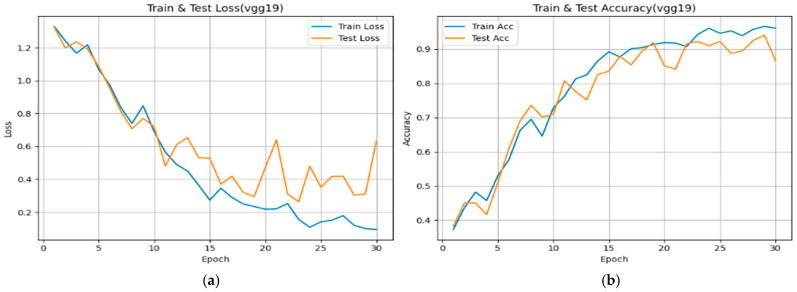
Training and validation performance curves showing the evolution of loss (**a**) and accuracy (**b**) over 30 epochs for the VGG19 model used in bladder cancer stage prediction.

**Figure 6:**
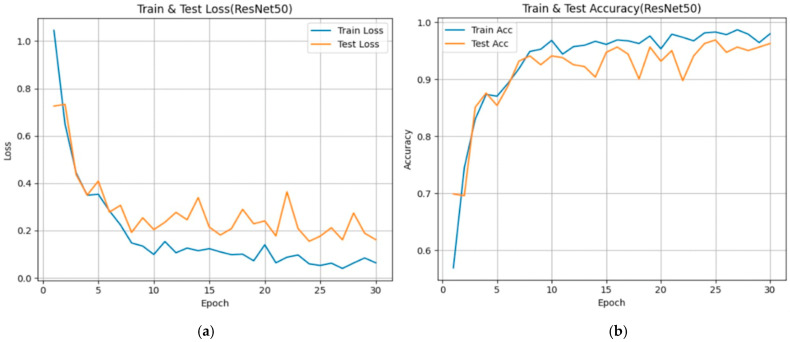
Training and validation performance curves showing the evolution of loss (**a**) and accuracy (**b**) over 30 epochs for the ResNet50 model used in bladder cancer stage prediction.

**Figure 7:**
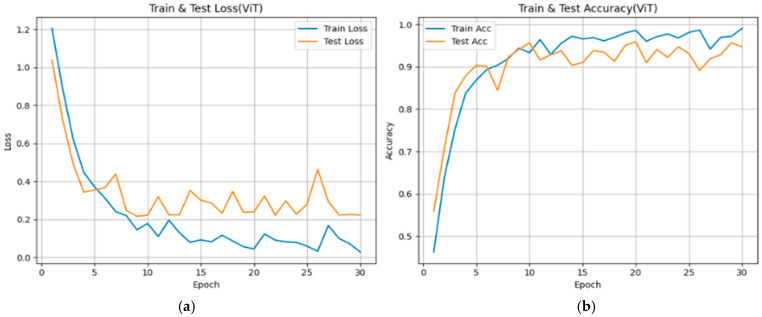
Training and validation performance curves showing the evolution of loss (**a**) and accuracy (**b**) over 30 epochs for the ViT model used in bladder cancer stage prediction.

From Figure 5, Figure 6 and Figure 7, each subfigure illustrates the evolution of loss and over 30 epochs for the training and validation datasets. Across all models, the training loss decreases steadily, while the validation loss shows convergence with minor fluctuations, indicating stable optimization behavior under the adopted training configuration. The corresponding accuracy curves exhibit rapid improvement during the early epochs (Epochs 0–10), followed by a plateau phase, suggesting convergence of the optimization process within the current experimental setting.

VGG19

The training and validation losses decline consistently, stabilizing after approximately 25 epochs. Validation accuracy reaches approximately 0.93–0.95, reflecting reliable performance within the present dataset, albeit with slightly higher variability compared to the other architectures. This behavior suggests effective learning of low- and mid-level image features, with moderate sensitivity to class imbalance and data variability.

ResNet50

The ResNet50 model exhibits smooth convergence of both loss and accuracy curves, with limited divergence between training and validation trajectories. Validation accuracy approaches approximately 0.97 under the current experimental design. These learning dynamics are consistent with the stabilizing effect of residual connections in facilitating optimization of deeper networks. However, performance should be interpreted as conditional on the adopted dataset and training pipeline, rather than as evidence of general or population-level superiority.

Vision Transformer (ViT) 

The ViT model demonstrates a gradual decline in training loss and validation accuracy comparable to the CNN-based architectures (approximately 0.94–0.96). Minor oscillations in validation loss are observed, which may reflect increased sensitivity to data variability given the relatively limited dataset size in relation to model complexity. Nonetheless, the ViT achieves stable convergence within the present experimental setting, indicating its capacity to capture global spatial relationships in MRI images.

Overall, all three models achieved consistently high training and validation performance without clear evidence of severe overfitting under the current experimental conditions. While the models demonstrated broadly comparable performance, ResNet50 was selected for subsequent illustrative analyses due to its stable optimization behavior and favorable calibration characteristics within the adopted pipeline.

**Figure 8:**
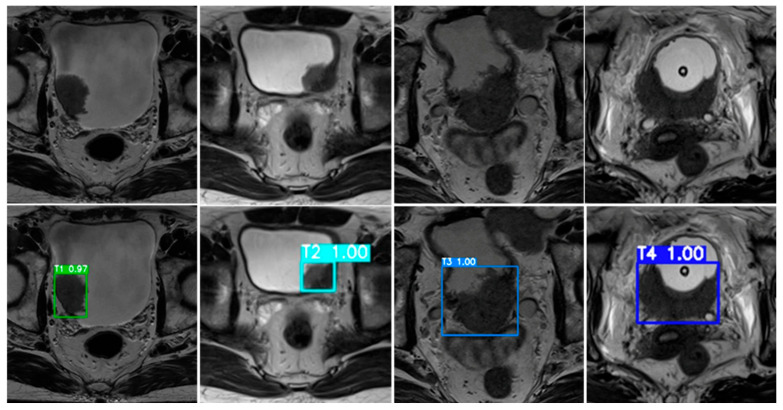
Representative MRI-based bladder cancer stage predictions generated by the proposed AI framework using the ResNet50 model—the best-performing classifier in this study.

To further illustrate model behavior, Figure 8 presents representative examples of bladder cancer stage predictions generated by the ResNet50 model. Each MRI slice corresponds to a VI-RADS–derived, MRI-based tumor stage category (T1–T4) assigned through expert radiological assessment. The upper row displays the original T2-weighted MRI images, while the lower row shows the corresponding model outputs, including bounding boxes and associated confidence scores.

In these representative cases, the proposed framework successfully localized tumor regions and generated stage predictions that were consistent with the reference MRI-based labels. The model demonstrates the ability to distinguish between non–muscle-invasive (T1–T2) and muscle-invasive (T3–T4) disease categories at the imaging level, reflecting its capacity to capture morphological and textural features relevant to VI-RADS–based staging. These examples are intended to illustrate qualitative consistency with radiological interpretation rather than to provide quantitative evidence of generalization or clinical decision-making performance.

Taken together, these findings support the feasibility of the proposed framework as a radiology-oriented system for automated replication and consistency assessment of MRI-based bladder cancer staging, rather than as a substitute for pathology-confirmed staging or definitive clinical decision-making.

From Figure 8, the upper row displays representative T2-weighted MRI slices corresponding to MRI-based tumor stage categories (T1–T4) derived from expert radiological assessment, while the lower row shows the corresponding model outputs with bounding boxes and associated confidence scores. In these illustrative cases, the model generated stage predictions that were consistent with the reference MRI-based labels. These examples demonstrate the model’s ability to differentiate between non–muscle-invasive (T1–T2) and muscle-invasive (T3–T4) disease categories at the imaging level. Importantly, these qualitative examples are intended to illustrate consistency with radiological interpretation rather than to provide quantitative evidence of generalization, diagnostic accuracy, or clinical decision-making performance.

To provide a more structured statistical characterization beyond point estimates, we evaluated performance variability, uncertainty, and probabilistic behavior using patient-level clustered bootstrap resampling, formal hypothesis testing, and calibration analysis. This evaluation framework is particularly relevant given the modest dataset size, the multi-class nature of MRI-based T1–T4 staging, and the use of high-capacity deep learning models. These analyses characterize uncertainty under the adopted experimental design and fixed learning pipeline.

Among the evaluated classifiers, ResNet50 exhibited stable optimization behavior and was therefore selected for more detailed statistical analysis within the adopted pipeline. Using 2000 patient-level bootstrap resamples, the model achieved an accuracy of 0.969 (95% CI: 0.947–0.984) and an AUC of 0.994 (95% CI: 0.985–0.999). These confidence intervals quantify the sampling uncertainty of performance estimates conditional on the fixed training and evaluation procedure and should not be interpreted as evidence of general or population-level superiority. Calibration analysis, assessed using the Brier score, yielded a value of 0.014 (95% CI: 0.008–0.021), indicating well-behaved probabilistic outputs under the current experimental setting.

To compare classification models under paired experimental conditions, formal statistical tests were conducted. Differences in AUC were evaluated using DeLong’s test, while differences in classification errors were assessed using McNemar’s test on paired predictions. These analyses provide a conditional comparison of model behavior within the present dataset and fixed evaluation pipeline, rather than evidence of generalizable performance ranking across architectures.

**Figure 9:**
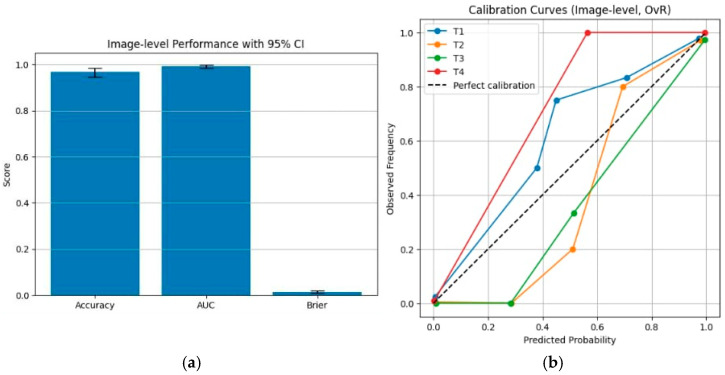
Image-level statistical performance and calibration analysis of the ResNet50 model. (a) Image-level classification performance evaluated using patient-level clustered bootstrap resampling, reporting accuracy, AUC, and Brier score with corresponding 95% confidence intervals. (b) Calibration curves for MRI-based tumor stages in a one-vs-rest setting, reconstructed using a coarser, sample-balanced binning strategy to provide a more stable qualitative depiction of probabilistic behavior under the current experimental setting.

The detailed bootstrap distributions, confidence intervals, and calibration characteristics are summarized in the accompanying tables and figures. In particular, Figure 9a illustrates bootstrap-based variability in accuracy and AUC estimates, while Figure 9b presents the calibration curve and associated reliability metrics for ResNet50. Together, these visualizations complement the numerical results by illustrating performance variability and probabilistic behavior under the current experimental setting and fixed learning pipeline, without implying generalization beyond the evaluated dataset.

These results indicate that, under the fixed training and evaluation pipeline adopted in this study, ResNet50 yields high point estimates of image-level discriminative performance with associated patient-level bootstrap confidence intervals that characterize sampling variability. Following revision of the calibration procedure using a coarser and sample-balanced binning strategy in the one-vs-rest multi-class setting, the calibration curves provide a clearer and more stable qualitative depiction of the relationship between predicted probabilities and observed outcome frequencies. Together, these analyses describe performance characteristics and probabilistic behavior conditional on the current experimental design, without implying robustness, generalization, or clinical reliability beyond the evaluated dataset.

## 4. Discussion

This study highlights the potential of deep learning–based frameworks to support MRI-based staging of bladder cancer through an integrated pipeline combining tumor localization, segmentation, and stage classification. Across the evaluated classification architectures, including ResNet50, VGG19, and Vision Transformer (ViT), all models demonstrated comparable and consistently high discriminative performance within the present dataset. While ResNet50 yielded slightly higher point estimates of accuracy and AUC under the adopted experimental setting, the observed performance differences across architectures were modest and should be interpreted cautiously, without implying general or population-level superiority of any specific model.

Although the point estimates of accuracy and AUC are high, the inclusion of patient-level bootstrap confidence intervals and calibration analysis primarily serves to characterize the uncertainty of performance estimates under the fixed learning and evaluation pipeline considered in this study. These intervals quantify variability induced by the adopted resampling scheme and should not be interpreted as evidence of generalization beyond the present dataset or under alternative data partitions. Accordingly, the reported results reflect stability within the current experimental design, while further validation using independent cohorts and fully repeated end-to-end resampling would be required to substantiate claims of broader generalizability. From a clinical standpoint, accurate MRI-based staging of bladder cancer is essential for risk stratification and radiological assessment, particularly in distinguishing between non–muscle-invasive (T1–T2) and muscle-invasive (T3–T4) disease categories. In the present study, both model training and evaluation rely on radiology-derived reference labels based on VI-RADS MRI staging, which reflect expert human interpretation rather than pathology-confirmed ground truth. Under this setting, the proposed framework should be interpreted as a feasibility study for automated replication and internal consistency assessment of VI-RADS–based MRI staging, rather than as an independent system capable of improving or superseding clinical decision-making. Accordingly, the model outputs represent automation of existing radiological judgments, with potential utility for standardization and workflow support, rather than evidence of added diagnostic value beyond the reference labeling process.

Before any clinical deployment, however, extensive iterative testing and validation are essential to ensure reliability, reproducibility, and safety. In particular, the model should undergo:

1) repeated evaluation on multi-center and heterogeneous datasets, encompassing different MRI scanners, imaging protocols, and patient populations;
2) cross-validation by independent radiologists, comparing AI-generated MRI-based stage assignments with expert radiological assessment guided by VI-RADS criteria as the imaging reference standard;
3) longitudinal performance monitoring to assess whether predictive behavior remains stable across time and evolving clinical settings. Such multi-phase evaluation is necessary to establish statistical robustness and to identify potential sources of bias prior to real-world integration.

The modular design of the proposed framework—based on a sequential pipeline for tumor localization, segmentation, and stage classification—enhances its adaptability for future clinical translation. A prospective development pathway includes the implementation of a secure, HIPAA- and GDPR-compliant web or mobile platform capable of accepting anonymized DICOM MRI inputs, performing automated tumor segmentation and MRI-based stage prediction, generating calibrated confidence scores for each predicted stage, and providing visual overlays to enhance physician interpretability and support collaborative decision-making within routine radiological workflows. Crucially, the development and refinement of such systems must occur in close collaboration with medical specialists. Continuous feedback loops involving radiologists and oncologists are fundamental to optimizing model performance, usability, and clinical acceptance. The predictions generated by the proposed system are intended to support—not replace—clinical expertise, ensuring that physicians remain central to diagnostic and therapeutic decision-making.

To advance toward clinical readiness, future work will focus on conducting prospective multi-institutional pilot studies to evaluate performance under real-world clinical workflows; integrating explainable AI (XAI) techniques, such as Grad-CAM, to highlight MRI regions contributing to classification decisions; incorporating histopathological confirmation to further strengthen stage labeling; and developing a continuous learning framework capable of updating model parameters as new validated clinical data become available. Together, these steps aim to ensure that the proposed AI system achieves not only high computational performance, but also statistical reliability, interpretability, and clinical trust, ultimately paving the way for responsible deployment as an AI-assisted MRI-based staging tool in bladder cancer care.

A limitation of this study is that model development and evaluation were conducted using a single publicly available dataset. Although this dataset provides standardized annotations and facilitates methodological benchmarking, it may not fully capture the heterogeneity of real-world clinical MRI data across institutions, scanner vendors, and acquisition protocols. In addition, data augmentation was performed prior to the train–test split, and augmented images were included in both the training and test sets. Consequently, augmented samples derived from the same original MRI slice may have been distributed across both sets, introducing within-sample correlation and potential information leakage. Under this experimental setting, the reported performance metrics may therefore partially reflect robustness to augmentation-based transformations rather than true generalization to unseen, original clinical MRI data, and should be interpreted with appropriate caution.

Furthermore, the present study does not include a dedicated quantitative evaluation of segmentation performance using overlap-based metrics (e.g., Dice coefficient or Intersection-over-Union). As a result, the reliability of the segmentation component is inferred indirectly through downstream classification outcomes, which represents an additional limitation. Although data augmentation was employed to mitigate class imbalance, imbalance among tumor stage categories remained. Synthetic augmentation may not fully capture the true anatomical variability and heterogeneity of bladder tumors observed in real-world clinical MRI data, and residual imbalance may therefore influence the observed performance. In parallel, this study relied on well-established standard architectures and did not explore task-specific network design or extensive architectural tuning tailored to bladder MRI characteristics. While such models provide strong and reproducible baselines, they may not fully exploit domain-specific features relevant to medical imaging.

Nevertheless, the inclusion of patient-level bootstrap confidence intervals and calibration analysis provides a more structured characterization of performance variability and probabilistic behavior for the fixed trained pipeline evaluated in this study. We emphasize that these analyses do not account for uncertainty arising from data-dependent preprocessing steps, such as ROI extraction, segmentation, or augmentation, which were not re-estimated within each resampling iteration. A fully rigorous assessment of generalization would require end-to-end retraining of all pipeline components within each resampled split, which was beyond the scope of the present work.

From a clinical translation perspective, while the proposed framework demonstrates promising technical performance, its current role should be framed as algorithmic support for consistency and feasibility assessment of MRI-based staging, rather than as an autonomous or independent decision-support system. Given the reliance on VI-RADS–based radiological labels, the predictions generated by the model should be regarded as structured reproductions of expert interpretation, intended to support radiological workflows and standardization efforts, rather than to directly influence clinical decision-making in the absence of independent validation.

Future investigations will therefore focus on implementing patient-level data splitting prior to augmentation; restricting evaluation to test sets composed exclusively of original MRI images; validating the framework on independent, multi-center cohorts encompassing diverse MRI scanners, imaging protocols, and patient populations; exploring complementary strategies for handling class imbalance (e.g., class-weighted or focal loss functions and patient-level resampling); investigating architectures optimized for medical image analysis (e.g., attention-guided CNNs, hybrid CNN–Transformer models, and self-supervised or multi-task learning frameworks); integrating explainable AI tools to improve transparency and clinical acceptance; and incorporating pixel-level ground-truth annotations with quantitative segmentation metrics to enable a more rigorous and independent assessment of segmentation accuracy and its contribution to the overall pipeline.

## 5. Conclusions

This study presents an integrated deep learning framework for MRI-based bladder cancer staging, combining YOLOv11 and DeepLabV3 for automated tumor localization and segmentation with multiple classification architectures, including VGG19, ResNet50, and Vision Transformer (ViT). Across the evaluated models, all architectures demonstrated comparable discriminative performance in differentiating MRI-based tumor stage categories (T1–T4) within the present dataset. While ResNet50 yielded slightly higher point estimates under the adopted experimental setting, these differences were modest and should be interpreted as descriptive rather than indicative of general or population-level superiority.

The inclusion of uncertainty quantification and calibration analysis provides a structured characterization of performance variability under the fixed learning and evaluation pipeline considered in this study. These analyses support the internal consistency of the reported results within the current experimental design, but do not constitute evidence of generalization beyond the present dataset or alternative data partitions. From a radiological perspective, the findings demonstrate the feasibility of applying artificial intelligence to automate and standardize MRI-based staging procedures derived from established VI-RADS interpretation, rather than to independently improve diagnostic accuracy.

The proposed framework offers a reproducible and objective approach for identifying tumor regions and assigning MRI-based stage categories derived from radiological reference labels. Under this design, the system should be interpreted as a feasibility study for automated replication and consistency assessment of VI-RADS–based MRI staging, with potential utility in supporting structured interpretation and workflow standardization, particularly in complex or borderline cases, rather than as an autonomous clinical decision-support system.

Nevertheless, before any clinical implementation, the proposed framework requires rigorous multi-phase validation to ensure reliability, reproducibility, and generalizability across diverse clinical environments. Future evaluations should include multi-center datasets encompassing heterogeneous MRI scanners, acquisition protocols, and patient populations, as well as independent assessment by expert radiologists using standardized imaging criteria. Where available, integration with pathology-confirmed staging may further strengthen validation and contextual interpretation.

With continued refinement and close collaboration between AI developers and medical specialists, the proposed framework may be translated into a radiology-oriented support application capable of processing anonymized DICOM MRI data, visualizing tumor segmentation overlays, and generating calibrated, MRI-based stage predictions. Importantly, this system is intended to support—rather than replace—physicians, serving as a complementary analytical tool that facilitates consistency and transparency in radiological assessment. Overall, this work establishes a methodological foundation for future studies aimed at developing clinically robust and ethically responsible AI-assisted tools for MRI-based bladder cancer staging.

## Figures and Tables

**Table 1:** Summary of original and augmented MRI images for each bladder cancer stage (T1–T4).

Bladder Cancer Stage	Original Image Count (Before Augmentation)	Number of Images (After Augmentation)
T1	272	531
T2	76	372
T3	41	365
T4	27	342
Total	416	1610

**Table 2:** Comparative performance of deep learning models for bladder cancer stage prediction.

Model	Accuracy	Precision				Recall				F1-Score			
		T1	T2	T3	T4	T1	T2	T3	T4	T1	T2	T3	T4
VGG19	0.94	0.90	0.95	0.94	1.00	0.95	0.93	0.93	0.94	0.93	0.94	0.94	0.97
ResNet50	0.96	0.95	1.00	0.94	0.99	0.96	0.97	0.96	0.99	0.96	0.97	0.96	0.99
ViT	0.94	0.90	0.95	0.94	1.00	0.95	0.93	0.93	0.94	0.93	0.94	0.94	0.97

## Data Availability

Bladder Cancer classification dataset from Kaggle is available on the Kaggle website (https://www.kaggle.com/datasets/shirtgm/bladder-cancer-classification, accessed on 1 September 2025).
